# Visualizing Aging

**DOI:** 10.1093/geroni/igaf122.1677

**Published:** 2025-12-31

**Authors:** Justine McGovern

**Affiliations:** CUNY--Lehman College, Bronx, New York, United States

## Abstract

This presentation aims to heighten awareness of ageism and provide alternatives to negative beliefs about older adults by offering a new look at lived experiences of aging. Justine has spent most of her career as a social work practitioner, educator and scholar exploring and expanding visual methods in gerontology practice, education and research. Now in her 60s, her many decades of experience documenting the lives of others in words and images and teaching students and colleagues how to engage with photography in social work practice, have taken a new turn. Borrowing from the humanities, she refers to herself as an imagistic phenomenologist. Her visual practice seeks to capture and communicate the life course of people, places and things, combining art, research, and advocacy to poke holes in deficit narratives about the passage of time and advanced age. Using her own images, Justine will discuss her process in becoming an artist later in life, as well as how to integrate a visual practice in gerontology education, research and practice.

